# Factors Associated with Findings of Published Trials of Drug–Drug Comparisons: Why Some Statins Appear More Efficacious than Others

**DOI:** 10.1371/journal.pmed.0040184

**Published:** 2007-06-05

**Authors:** Lisa Bero, Fieke Oostvogel, Peter Bacchetti, Kirby Lee

**Affiliations:** 1 Clinical Pharmacy and Health Policy, University of California, San Francisco, California, United States of America; 2 Department of Mathematics, University of Leiden, The Netherlands; 3 Department of Epidemiology and Biostatistics, University of California, San Francisco, California, United States of America; 4 Department of Clinical Pharmacy, University of California, San Francisco, California, United States of America; Italian Cochrane Centre, Italy

## Abstract

**Background:**

Published pharmaceutical industry–sponsored trials are more likely than non-industry-sponsored trials to report results and conclusions that favor drug over placebo. Little is known about potential biases in drug–drug comparisons. This study examined associations between research funding source, study design characteristics aimed at reducing bias, and other factors that potentially influence results and conclusions in randomized controlled trials (RCTs) of statin–drug comparisons.

**Methods and Findings:**

This is a cross-sectional study of 192 published RCTs comparing a statin drug to another statin drug or non-statin drug. Data on concealment of allocation, selection bias, blinding, sample size, disclosed funding source, financial ties of authors, results for primary outcomes, and author conclusions were extracted by two coders (weighted kappa 0.80 to 0.97). Univariate and multivariate logistic regression identified associations between independent variables and favorable results and conclusions. Of the RCTs, 50% (95/192) were funded by industry, and 37% (70/192) did not disclose any funding source. Looking at the totality of available evidence, we found that almost all studies (98%, 189/192) used only surrogate outcome measures. Moreover, study design weaknesses common to published statin–drug comparisons included inadequate blinding, lack of concealment of allocation, poor follow-up, and lack of intention-to-treat analyses. In multivariate analysis of the full sample, trials with adequate blinding were less likely to report results favoring the test drug, and sample size was associated with favorable conclusions when controlling for other factors. In multivariate analysis of industry-funded RCTs, funding from the test drug company was associated with results (odds ratio = 20.16 [95% confidence interval 4.37–92.98], *p* < 0.001) and conclusions (odds ratio = 34.55 [95% confidence interval 7.09–168.4], *p* < 0.001) that favor the test drug when controlling for other factors. Studies with adequate blinding were less likely to report statistically significant results favoring the test drug.

**Conclusions:**

RCTs of head-to-head comparisons of statins with other drugs are more likely to report results and conclusions favoring the sponsor's product compared to the comparator drug. This bias in drug–drug comparison trials should be considered when making decisions regarding drug choice.

## Abstract 2

Lisa Bero and colleagues found published trials comparing one statin with another were more likely to report results and conclusions favoring the sponsor's product than the comparison drug.

## Editors' Summary

**Background.:**

Randomized controlled trials are generally considered to be the most reliable type of experimental study for evaluating the effectiveness of different treatments. Randomization involves the assignment of participants in the trial to different treatment groups by the play of chance. Properly done, this procedure means that the different groups are comparable at outset, reducing the chance that outside factors could be responsible for treatment effects seen in the trial. When done properly, randomization also ensures that the clinicians recruiting participants into the trial cannot know the treatment group to which a patient will end up being assigned. However, despite these advantages, a large number of factors can still result in bias creeping in. Bias comes about when the findings of research appear to differ in some systematic way from the true result. Other research studies have suggested that funding is a source of bias; studies sponsored by drug companies seem to more often favor the sponsor's drug than trials not sponsored by drug companies

**Why Was This Study Done?:**

The researchers wanted to more precisely understand the impact of different possible sources of bias in the findings of randomized controlled trials. In particular, they wanted to study the outcomes of “head-to-head” drug comparison studies for one particular class of drugs, the statins. Drugs in this class are commonly prescribed to reduce the levels of cholesterol in blood amongst people who are at risk of heart and other types of disease. This drug class is a good example for studying the role of bias in drug–drug comparison trials, because these trials are extensively used in decision making by health-policy makers.

**What Did the Researchers Do and Find?:**

This research study was based on searching PubMed, a biomedical literature database, with the aim of finding all randomized controlled trials of statins carried out between January 1999 and May 2005 (reference lists also were searched). Only trials which compared one statin to another statin or one statin to another type of drug were included. The researchers extracted the following information from each article: the study's source of funding, aspects of study design, the overall results, and the authors' conclusions. The results were categorized to show whether the findings were favorable to the test drug (the newer statin), inconclusive, or not favorable to the test drug. Aspects of each study's design were also categorized in relation to various features, such as how well the randomization was done (in particular, the degree to which the processes used would have prevented physicians from knowing which treatment a patient was likely to receive on enrollment); whether all participants enrolled in the trial were eventually analyzed; and whether investigators or participants knew what treatment an individual was receiving.

One hundred and ninety-two trials were included in this study, and of these, 95 declared drug company funding; 23 declared government or other nonprofit funding while 74 did not declare funding or were not funded. Trials that were properly blinded (where participants and investigators did not know what treatment an individual received) were less likely to have conclusions favoring the test drug. However, large trials were more likely to favor the test drug than smaller trials. When looking specifically at the trials funded by drug companies, the researchers found various factors that predicted whether a result or conclusion favored the test drug. These included the impact of the journal publishing the results; the size of the trial; and whether funding came from the maker of the test drug. However, properly blinded trials were less likely to produce results favoring the test drug. Even once all other factors were accounted for, the funding source for the study was still linked with results and conclusions that favored the maker of the test drug.

**What Do These Findings Mean?:**

This study shows that the type of sponsorship available for randomized controlled trials of statins was strongly linked to the results and conclusions of those studies, even when other factors were taken into account. However, it is not clear from this study why sponsorship has such a strong link to the overall findings. There are many possible reasons why this might be. Some people have suggested that drug companies may deliberately choose lower dosages for the comparison drug when they carry out “head-to-head” trials; this tactic is likely to result in the company's product doing better in the trial. Others have suggested that trials which produce unfavorable results are not published, or that unfavorable outcomes are suppressed. Whatever the reasons for these findings, the implications are important, and suggest that the evidence base relating to statins may be substantially biased.

**Additional Information.:**

Please access these Web sites via the online version of this summary at http://dx.doi.org/10.1371/journal.pmed.0040184.

- The James Lind Library has been created to help people understand fair tests of treatments in health care by illustrating how fair tests have developed over the centuries
- The International Committee of Medical Journal Editors has provided guidance regarding sponsorship, authorship, and accountability
- The CONSORT statement is a research tool that provides an evidence-based approach for reporting the results of randomized controlled trials
- Good Publication Practice guidelines provide standards for responsible publication of research sponsored by pharmaceutical companies
- Information from Wikipedia on Statins. Wikipedia is an internet encyclopedia anyone can edit

## Introduction

Bias is the combination of various study design, data analysis, and presentation factors that make the results differ systematically from the truth [1]. Various factors can lead to bias in randomized controlled trials (RCTs) of drug efficacy, including framing of the research question, design and conduct of the study, and analysis of the data [2,3]. Whether the results are reported in full, or whether there is selective reporting of outcomes can also contribute to biased results and conclusions [4–7].

Most studies that have attempted to identify factors that may be associated with bias have examined individual design features, including randomization [8,9], concealment of allocation [10], double blinding [9,10], sample size [11–14], choice of drug comparator [15–18], and choice of statistical analysis [19,20]. In this study, we examine the relative contributions of different factors to favorable results and conclusions.

One factor that has been associated with possible bias is funding source for a study. Trials supported by pharmaceutical companies are more likely than those with non-industry sponsors to report results and conclusions that are favorable towards the sponsor's product compared to placebo [14,15,21–28]. However, few studies examining the association of funding source and outcome adjust for potential confounders, such as study design characteristics or type of intervention. For example, study characteristics such as randomization sequence generation, concealment of allocation, blinding, sample size, or choice of drug comparator might also contribute to statistically significant results [28,29]. Although the association of pharmaceutical industry sponsorship with results and conclusions that favor the sponsor's product over placebo is clear [22,23], the potential influence of funding source when the funder manufactures one of two competing drugs undergoing comparison has not been well described. Although Heres et al. found that results of head-to-head comparisons of second-generation antipsychotics have contradictory conclusions depending on which company sponsored the study, they did not analyze the potential effects of other study design characteristics [30].

Statins are an interesting class of drug for investigating the influence of funding source on outcomes of head-to-head drug comparisons because a number of statins are manufactured by competing companies. Statins are widely prescribed as effective first-line agents for lowering cholesterol and other lipids. At the time of this study, seven statin drugs were marketed in the United States by competing companies, although one of these drugs has since been withdrawn. Alternative classes of drugs were also available to treat the same condition. As strong evidence suggests that statins are more effective than placebo in reducing lipids [31], drug–drug comparison trials involving statins are the most relevant for making policy decisions about choosing a statin. Choice of a statin should depend on data from statin–statin comparisons of low-density lipoprotein reduction at comparable doses, ability to achieve cholesterol-reduction goals, and effects on a variety of other outcomes such as death, coronary events, or stroke [32]. For example, formulary committees use head-to-head drug comparisons to decide which of the statins will be placed on their formulary. Therefore, it is important to explore possible biases in statin–statin comparisons.

This cross-sectional study examines associations between research funding source, study design characteristics aimed at reducing bias, and other factors for which results and conclusions have been published in RCTs of statin–drug comparisons. We hypothesized that the results and conclusions of trials are more likely to favor the statin made by the sponsor of the study and that other design features, such as concealment of allocation, blinding, and sample size are also associated with statistically significant results that favor the statin produced by the study's sponsor.

## Methods

### Search Strategy

We electronically searched PubMed to identify reports of RCTs published between January 1999 and May 2005. The following MeSH terms or Substance Names of the seven available statins were used: “simvastatin” OR “cerivastatin” OR “pravastatin” OR “atorvastatin” OR “fluvastatin” OR “rosuvastatin” OR “lovastatin”. The search was limited to “randomized controlled trials” and “humans”. We restricted our search to these years because journals strengthened their policies requiring disclosure of funding sources and financial ties of authors during this period [33]. We also searched the reference lists of all potentially relevant articles identified through the PubMed search. Our search included articles published in any language.

### Inclusion and Exclusion Criteria

We reviewed abstracts of all citations and retrieved articles based on the following inclusion criteria: (1) RCT; (2) statin drug compared to a different statin drug or another, non-statin, drug; (3) efficacy measured in humans; and (4) original research, defined as studies that appeared to present original data and did not specifically state that they were reviews. Studies with the primary objective of assessing the effect of a combination of a statin and another drug were included if there was a comparison of the statin alone with the other drug. If a placebo arm was also included in the trial, we included only the data from the statin–drug comparison.

The following exclusion criteria were used to screen all abstracts: (1) pharmacokinetic or pharmacodynamic studies, since they do not involve testing of clinical efficacy outcomes; (2) studies including only rationale and design elements, editorials, letters to the editor, commentaries, abstracts, unpublished reports, reviews; (3) studies comparing different doses of one type of statin; (4) studies comparing statins to placebo only; (5) studies comparing statins to a non-drug intervention (e.g., diet, exercise); (6) studies in which the statin was present in all the comparison groups; (7) absence of statistical comparison or lack of sufficient data; and (8) in vitro analyses. Any discrepancies about inclusion were discussed by the authors of the present paper until consensus was achieved. No identical publications were identified. However, as we were interested in the published reports from trials, we did include multiple publications from the same study if the publications reported different outcomes.

### Data Extraction

One investigator (F. Oostvogel), who was not blinded to author names and affiliations, funding sources, and financial disclosure, extracted all data from each article. A second coder (L. Bero), who was blinded to funding source and financial tie information, independently extracted data on concealment of allocation, selection bias, blinding, sample size, results for primary outcomes, and author conclusions. Inter-coder reliability was very good (weighted kappa 0.80 to 0.97). In cases of disagreement, the two coders discussed the papers and reached agreement. We extracted data on the following publication characteristics, which have been shown to be independently related to favorable results or conclusions of drug studies [8–10,13,22,27,34,35].

### Journal Characteristics

#### Peer-review status.

Each article was classified as peer reviewed, non-peer reviewed, or unknown, based on information found on the website of the journal where the article was published. A publication was considered peer reviewed if the website mentioned that the journal had a peer-review process or if it was stated that the manuscripts were evaluated by at least one external expert in the field; otherwise, a publication was considered non-peer reviewed. Peer-review status was classified as unknown if we could find no information on the journal.

#### Impact factor.

Impact factor was obtained from the Institute for Scientific Information, 2004 data [36].

### Author Characteristics

#### Institutional affiliation.

The institutional affiliation of the corresponding author was obtained from the article and classified into (1) academic/university, (2) government, (3) private nonprofit, (4) industry, (5) hospital, (6) other, or (7) unable to determine.

#### Country of origin.

The country of origin of the corresponding author was recorded and categorized into low income, lower-middle income, upper-middle income, and high income economies based on the World Bank Group classifications [37].

### Study Design Characteristics

#### Study design.

The study design for each article was classified as parallel or cross-over trial. Specific drugs being compared were recorded.

#### Comparison group.

The comparisons for the primary outcome were classified as (1) statin versus statin or (2) statin versus other drug. In statin-versus-statin comparisons, the “test” drug was defined as the newest statin (most recent FDA approval date) and the older statin as the “comparator” drug. In statin-versus-other-drug comparisons, the “test” drug was defined as the statin and the other drug as the “comparator” drug.

#### Type of primary outcome measure.

The primary outcome measured was classified as (1) surrogate if the end point was a marker for a clinical event (e.g., lipid levels, artery diameter, endothelial function) or (2) clinical if a real clinical event (e.g., stroke, myocardial infarction, death) was measured.

#### Sample size.

We recorded the number of patients that were included in the analyses.

#### Primary results.

For each published paper, the result reported for each primary outcome was categorized as (1) favorable if the result was statistically significant (*p* < 0.05 or confidence interval [CI] excluding no difference) and in the direction of the test drug being more efficacious or less harmful (in the case of side effects); (2) inconclusive if the result did not reach statistical significance; or (3) unfavorable if the result was statistically significant in the direction of the comparator drug being more efficacious or less harmful. If a study explicitly stated that it was designed as a non-inferiority study and the two comparisons drugs were equivalent, the result was coded as favorable.

The entire set of results for all primary outcomes in each paper was then classified as favorable if at least one primary outcome was favorable and none were unfavorable; otherwise, the entire set was classified as unfavorable.

#### Conclusion.

The conclusions reported in the published papers were categorized as (1) favorable if the test drug was preferred to comparator; (2) about equal if the test drug was about equal to comparator; or (3) not favorable if the comparator drug was preferred to the test drug. If a study explicitly stated that it was designed as a non-inferiority study and the two drugs being compared were equivalent, the conclusion was coded as favorable. If an article did not clearly state that one of the two drugs was better or if the two drugs had different advantages, the conclusion was coded as “test drug about equal to comparator”. For analysis, conclusions were categorized as favorable or not favorable (combining about equal and not favorable).

### Funding Information

#### Funding source.

The funding source of each published study was categorized as (1) industry, (2) private nonprofit, (3) government, (4) other, (5) multiple sources, (6) no funding, and (7) none disclosed. For analysis, the funding-source categories were collapsed into (1) industry, (2) none disclosed/no funding, and (3) government/private nonprofit.

#### Financial ties.

Data about the financial ties of each author were extracted and coded for (1) whether or not there were any financial ties disclosed with the sponsor of the study and (2) whether or not there were any financial ties disclosed with any other company (yes, no, or none disclosed).

#### Role of the sponsor.

Information about the role of the sponsor was coded as (1) role of sponsor not mentioned, (2) sponsor not involved in study design and analyses, (3) sponsor involved, or (4) no sponsor involved.

### Study Design Characteristics Aimed at Reducing Bias

Studies that met the inclusion criteria were rated for study design features according to the components reported by Chalmers et al. [38]. Chalmers used three different categories: method of treatment assignment (randomization and concealment of allocation), control for whether all participants enrolled in the trial have been included in the analysis (intention-to-treat analysis and loss to follow-up), and blinding of participants and investigators. For each category, the score can range from 0 to 3, where higher scores indicate better methodological quality. For analysis, we dichotomized each category into “adequate” (score of 2 or 3) or “inadequate” (score of 0 or 1).

### Statistical Analysis

We report the frequency of the different characteristics of each article. For characteristics where there was sufficient variability, we analyzed the characteristics by the direction of results and conclusions to determine whether certain characteristics were associated with favorable results or conclusions. Proportions of manuscripts with favorable results or conclusions were first analyzed using univariate logistic regression and estimating odds ratios (ORs) to identify associations between independent variables and favorable results and conclusions. Although impact factor and sample size were continuous variables, they were modeled categorically because their effects were clearly nonlinear.

To control for multiple variables simultaneously, we carried out multivariate logistic regression analysis and calculated ORs. These models included funding source and all factors that had *p* < 0.05 in univariate models for either favorable results or conclusions. For our primary analysis, we conducted the regression analyses on our full sample (*n* = 192). For our a priori analysis of drug industry–sponsored studies, we conducted the regression analyses on the subsample of studies that were industry funded (*n* = 95) in order to examine the association between funding from the test drug company and results or conclusions that favor the test drug.

Our target sample size was to have 40 trials that had results or conclusions favoring the test drug. We chose this sample size so there would be at least ten trials with favorable results or conclusions per predictor in a multivariate analysis with up to four simultaneous predictors. We achieved this target sample size for both the full sample and subsample of industry-funded trials. Data were analyzed with SAS software (version 9.1, SAS Institute, Cary, North Carolina, United States).

## Results

### Characteristics of Included Studies

**Figure 1:**
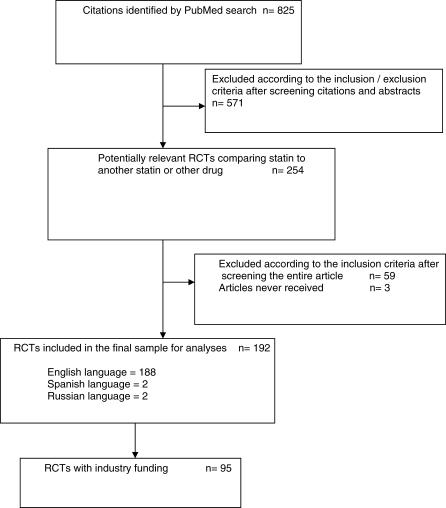
Flowchart of Manuscript Selection

Our final sample consisted of 192 published RCTs (see Figure 1). The characteristics of the full sample are shown in Table 1, and the full list of references to included studies is presented in Table S1. Almost all (98%, 189/192) studies reported only surrogate outcome measures. There was also little variability in the peer-review status, study design, or country of origin or institutional affiliation of corresponding authors. Therefore, these variables were not included in our regression analyses. Impact factor and sample size were divided into quartiles with the upper three quartiles compared with the lowest quartile. Forty-nine percent of articles had conclusions that favored the test drug, 15% had a conclusion that favored the comparator drug, and 36% concluded that the two drugs were about equal. Of the 192 included trials, 95 (49%) disclosed funding from industry sponsors.

**Table 1:**
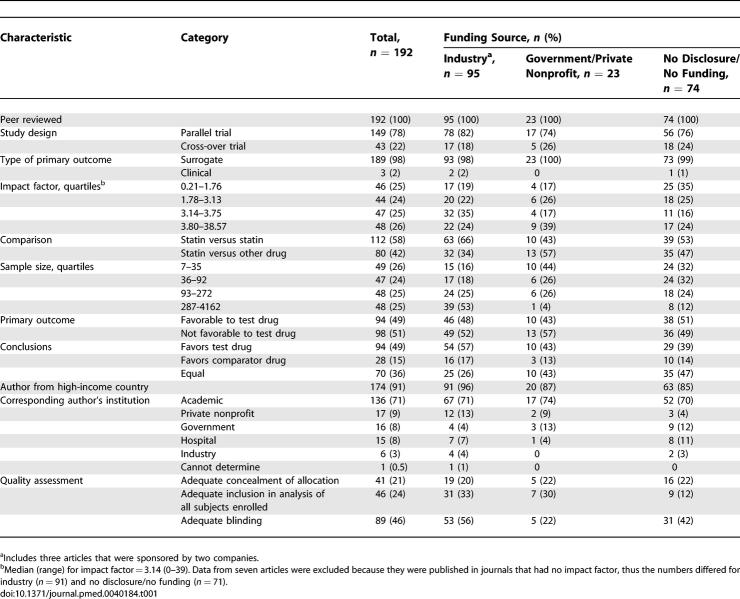
Characteristics of Included Articles by Funding Source (*n* = 192)

Among the 95 articles declaring industry funding, the role of the sponsor was disclosed in 20 (21%) of these. One trial stated that the sponsor was not involved in the study design and analyses, and 19 trials stated that the sponsor was involved by providing the study drug, data analyses, or writing and preparation of the manuscript.

### Analysis of Full Sample

Table 2 shows the results of univariate logistic regression analyses. Studies with adequate blinding were substantially less likely to report results favoring the test drug than studies that did not include adequate blinding. Trials with larger sample sizes were more likely to report conclusions that favored the test drug, while trials with no disclosed funding sources were less likely to have conclusions favoring the test drug compared to trials with industry funding. In multivariate analyses, trials with adequate blinding remained significantly less likely to report statistically significant results favoring the test drug, and sample size remained associated with favorable conclusions when controlling for other factors (Table 3). Pooling non-industry-funded studies and those with no funding disclosure produced ORs of 1.49 (95% CI 0.75–3.0, *p* = 0.26) for results and 0.73 (95% CI 0.36–1.46, *p* = 0.37) for conclusions versus industry-funded studies. Adding interaction terms for industry funding versus all others with sample size quartile did not produce a statistically significant improvement in the fit to the data for results (*p* = 0.21 by likelihood ratio test) and also did not show a consistent pattern (OR of all others 2.1, 1.15, 3.3, 0.42 in first to fourth sample size quartiles, respectively). For conclusions, the interaction terms also did not reach statistical significance overall (*p* = 0.11), but the pattern was in a consistent direction (OR of all others 2.2, 1.02, 0.52, 0.18 in first to fourth sample size quartiles, respectively).

**Table 2:**
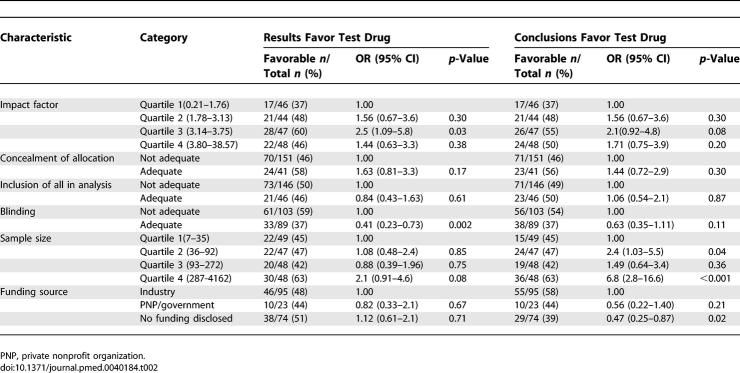
Association between Characteristics of Articles (*n* = 192) and Statistically Significant Results or Conclusions that Favor the Test Drug: Univariate Logistic Regression

**Table 3:**
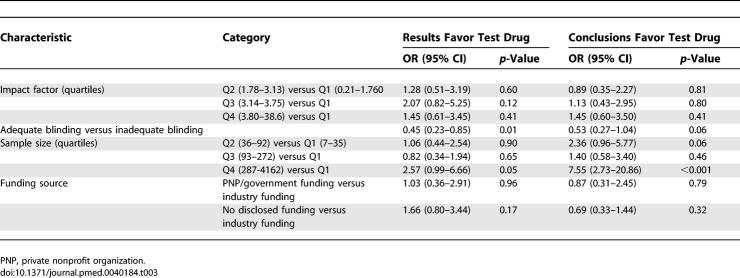
Association between Characteristics of Manuscripts (*n* = 192) and Results or Conclusions that Favor Test Drug: Multivariate Analysis

We also conducted a multivariate analysis for the subset of articles that were statin–statin comparisons (*n* = 112), and the results were essentially the same as for the comparisons between statin and any non-statin or statin drug. Trials with adequate blinding remained significantly less likely to report statistically significant results favoring the test drug (OR = 0.28 [95% CI 0.11–0.73], *p* = 0.0095), and sample size remained associated with favorable conclusions (OR = 8.49 [95% CI 1.93–37.36], *p* = 0.0047) when controlling for other factors.

The data used in the analyses are presented in Tables S2 and S3.

### Analysis of Industry-Sponsored Trials

In univariate logistic regression analyses of the industry-sponsored trials, higher impact factor, larger sample size, and funding from the test drug company were associated with favorable results, while trials with adequate blinding were less likely to report statistically significant results favoring the test drug (Table 4). Larger sample size and funding from the test drug company were also associated with favorable conclusions (Table 4). In multivariate logistic regression analysis, funding from the test drug company remained associated with statistically significant results favoring the test drug (OR = 20.16 [95% CI 4.37–92.98], *p* < 0.001) or conclusions favoring the test drug (OR = 34.55 [95% CI 7.09–168.4], *p* < 0.001) (Table 5) when controlling for other factors. Studies with adequate blinding remained less likely to report statistically significant results favoring the test drug (Table 5). Adding interaction terms for funding by test drug company with sample size quartile did not produce a statistically significant improvement in the fit to the data for results (*p* = 0.38), although the ORs associated with test drug company funding did show an increasing pattern (3.8, 8.1, infinite, 59.5 in the first to fourth sample size quartiles, respectively). For conclusions, the interaction *p*-value was *p* = 0.066, with ORs associated with test drug company funding of infinite, 2.1, infinite, and 143.2 in the first to fourth sample size quartiles, respectively. The infinite estimated ORs result from no favorable outcomes for some combinations of funding and sample size quartile, making these results difficult to interpret.

**Table 4:**
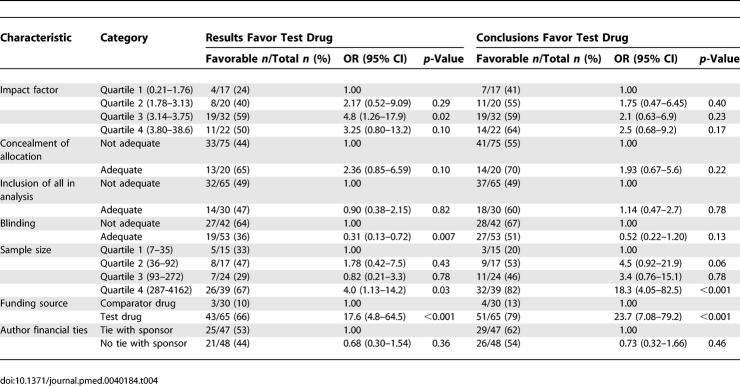
Association between Characteristics of Industry-Funded Articles (*n* = 95) and Statistically Significant Results and Conclusions that Favor the Test Drug: Univariate Logistic Regression

**Table 5:**
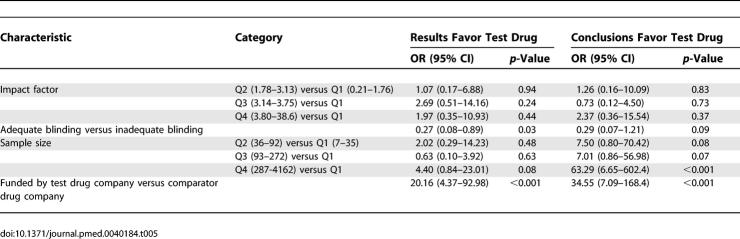
Association between Characteristics of Industry-Funded Manuscripts (*n* = 95) and Results or Conclusions that Favor Test Drug: Multivariate Analysis

We also conducted the multivariate logistic regression analysis for the subset of industry-funded studies that were statin–statin comparisons (*n* = 63), and the results were essentially the same as for the comparisons between statin and any non-statin or statin drug. Funding from the test drug company remained associated with statistically significant results favoring the test drug (OR = 16.06 [95% CI 2.22–116.3], *p* = 0.043) or conclusions favoring the test drug (OR = 77.09 [95% CI 7.92–749.9], *p* < 0.001) when controlling for other factors.

## Discussion

We examined the association between study design characteristics and the results and conclusions of RCTs of head-to-head comparisons of statins with other drugs. We hypothesized that the results and conclusions of published trials would be more likely to favor the statin made by the sponsor of the study and that other design features, such as concealment of allocation, blinding, and sample size, would also be associated with results or conclusions that favor the statin. We found that the main factor associated with the results and conclusions of industry-sponsored research to compare statin drugs with statin or non-statin drugs is research sponsorship. Our study adds new information to the body of literature showing that pharmaceutical industry–sponsored studies comparing drug and placebo are more likely to favor the drug [14,21–23,25,27,28,39]. Our finding suggests that favorable results and outcomes are associated with the specific sponsor of a study, even when all the studies are industry funded. This finding may help explain why well-designed head-to-head comparisons of statins and other drugs sometimes have contradictory results.

There are several possible explanations for our finding of the strong association between funding source and outcomes that are favorable to the drug company sponsor. First, it is possible that pharmaceutical companies selectively fund trials on drugs that are likely to produce a statistically significant result. This can be accomplished by selecting nonequivalent doses of drugs for testing [15–17]. A recent review of 42 RCTs comparing the low-density lipoprotein–lowering ability of two or mores statins found that almost all of the trials compared nonequivalent doses of statins [32]. Second, as we examined only published studies, publication bias, or the phenomenon of statistically significant results being published more frequently than statistically nonsignificant results, may explain the association of funding and outcome [40]. Selective reporting of outcomes can also contribute to biased results and conclusions [4–6]. In addition, industry sponsorship may be associated with multiple reporting of studies with favorable findings, emphasizing the imbalance towards statistically significant results in the published literature [18,41,42]. Finally, more than one third of the studies in our sample had no disclosed sponsorship. It is possible that industry funders or industry-supported authors could fail to disclose the sponsorship of a published study if the findings do not support the sponsor's product. This, however, would have to be very prevalent to explain by itself the results in Tables 4 and 5 concerning sponsorship. For example, nearly all the 29 studies with favorable conclusions and undisclosed funding would have to be actually funded by the comparator drug company, and nearly all the 45 studies without favorable conclusions would have to be funded by the test drug company in order to erase the difference shown in Table 4.

We identified a number of weaknesses common to published statin versus statin or non-statin drug comparisons that bring into question their clinical relevance. The most important weakness was the lack of patient-related clinical-outcome measures. Looking at the totality of available evidence, we found that almost all studies (98%, 189/192) used only surrogate outcome measures. Inadequate blinding, lack of concealment of allocation, poor follow-up, and lack of intention-to-treat analyses were common among these studies. These weaknesses suggest that these types of studies should be used with great caution by those making regulatory and purchasing decisions.

We found that adequate double blinding was an influential design feature in our sample. Adequately blinded studies were less likely to report results favoring the sponsor's product. Although RCTs of poorer quality are more likely to reach biased conclusions [10,43,44], the unreliability of quality scores has led to the recommendation that trial quality be assessed for individual features that are aimed to reduce bias, such as concealment of allocation or blinding [45]. Recent research further suggests that specific study design characteristics are not reliably associated with treatment effect sizes across different studies and medical areas [46]. Thus, the study design characteristics associated with statistically significant results might vary with the type of research being examined. We used three items from the Chalmers et al. [38] methodological quality assessment scale to assess study design features that might be associated with results and conclusions. These items focus on important aspects of trial design: (1) concealment of allocation, (2) control for whether all patients enrolled in the trial were included in the analysis (drop outs and intention-to-treat analyses), and (3) blinding of participants and investigators. Previous studies showed that these specific characteristics are associated with bias in clinical trials [10,47,48]. For example, Schulz and colleagues found that estimates of treatment effects were exaggerated by 41% for inadequately concealed trials and by 17% for trials with inadequate double blinding [10].

Finally, journal characteristics may influence the results and conclusions of articles as the quality of the reporting may vary with the journal. For example, articles published in peer-reviewed journals have superior quality compared to articles published in non-peer-reviewed journals [15,27]. In our sample, we had no variability in peer-review status, but we did observe a small possible association between journal impact factors and results and conclusions that favored the test drug.

Our study has several limitations, including our ability to identify funding sources and financial ties. We categorized studies as industry funded or not based on each article's disclosure of a trial's funding source(s). Krimsky showed, however, that there is a lack of disclosure of industry research support and personal financial ties across a wide variety of journals [49,50]. Thus, we may be underestimating the number of industry-sponsored studies and personal financial ties of investigators.

The market for statins is competitive. The National Cholesterol Education Program update of the Adult Treatment Panel III guidelines expanded both the scope and intensity of low-density lipoprotein–lowering therapy for prevention of cardiovascular disease [51]. To achieve the goals in the guideline, millions of Americans would need to be placed on cholesterol-lowering medication in higher doses and for a longer period, thereby increasing the number of prescriptions for statin drugs [51,52]. Eight of the nine members of the National Cholesterol Education Program panel had financial ties with pharmaceutical companies that manufactured statin drugs [51,52]. Our findings suggest that available data on choosing between statins based on head-to-head comparisons may also be influenced by financial conflicts of interest. Our findings may be generalizable to other classes of drugs with competitive markets.

There is increasing concern that the funding source influences outcomes and conclusions of medical research [3]. At the same time, industry support of biomedical research has increased dramatically during the past few decades [53,54]. The growing proportion of industry-funded studies could shift the balance of published trials more towards studies that favor new drugs [55]. This trend and our finding that, for one class of drugs, the results and conclusions of trials tend to favor the drug that is made by the sponsor raises important considerations for selecting drugs within a class. Sponsorship bias, even when controlling for other confounding study characteristics, may be the main explanation for contradictory findings of drug–drug comparison trials. This bias in drug–drug comparison trials should be considered when making health-policy decisions regarding drug choice, such as drug formulary decisions. Reviewers of published reports that disclose funding by the makers of the product being tested should be more critical of the methods than if the reports are not industry sponsored [56]

## Supporting Information

Table S1 Included Studies Table (62 KB PDF)

Table S2 Statin Data: Adjudicated Full and Subsample (105 KB XLS)

Table S3 Statin-Only Comparison Data (48 KB XLS)

## Abbreviations

CI: confidence interval
OR: odds ratio
RCT: randomized controlled trial
