# Supplementary material for: Factors Associated with Findings of Published Trials of Drug–Drug Comparisons: Why Some Statins Appear More Efficacious than Others
Source: PLoS Med. 2007 Jun 5;4(6):e184. doi: 10.1371/journal.pmed.0040184 (PMC1885451; doi:10.1371/journal.pmed.0040184)
Supplement: Supplementary file 1 [file pmed.0040184.st001.pdf]

Included Studies

1. Effect of nifedipine and cerivastatin on coronary endothelial function in patients with coronary artery disease: the ENCORE I Study (Evaluation of Nifedipine and Cerivastatin On Recovery of coronary Endothelial function). Circulation 2003;107(3):422-8.
2. Andrews TC, Ballantyne CM, Hsia JA, Kramer JH. Achieving and maintaining National Cholesterol Education Program low-density lipoprotein cholesterol goals with five statins. Am J Med 2001;111(3):185-91.
3. Asztalos BF, Horvath KV, McNamara JR, Roheim PS, Rubinstein JJ, Schaefer EJ. Comparing the effects of five different statins on the HDL subpopulation profiles of coronary heart disease patients. Atherosclerosis 2002;164(2):361-9.
4. Athyros VG, Papageorgiou AA, Athyrou VV, Demitriadis DS, Kontopoulos AG. Atorvastatin and micronized fenofibrate alone and in combination in type 2 diabetes with combined hyperlipidemia. Diabetes Care 2002;25(7):1198-202.
5. Athyros VG, Papageorgiou AA, Athyrou VV, Demitriadis DS, Pehlivanidis AN, Kontopoulos AG. Atorvastatin versus four statin-fibrate combinations in patients with familial combined hyperlipidaemia. J Cardiovasc Risk 2002;9(1):33-9.
6. Badia X, Russo P, Attanasio E. A comparative economic analysis of simvastatin versus atorvastatin: results of the Surrogate Marker Cost-Efficacy (SMaC) study. Clin Ther 1999;21(10):1788-96.
7. Bairaktari ET, Tzallas CS, Tsimihodimos VK, Liberopoulos EN, Miltiadous GA, Elisaf MS. Comparison of the efficacy of atorvastatin and micronized fenofibrate in the treatment of mixed hyperlipidemia. J Cardiovasc Risk 1999;6(2):113-6.
8. Ballantyne CM, Blazing MA, Hunninghake DB, Davidson MH, Yuan Z, DeLucca P, et al. Effect on high-density lipoprotein cholesterol of maximum dose simvastatin and atorvastatin in patients with hypercholesterolemia: results of the Comparative HDL Efficacy and Safety Study (CHESS). Am Heart J 2003;146(5):862-9.
9. Ballantyne CM, Blazing MA, King TR, Brady WE, Palmisano J. Efficacy and safety of ezetimibe co-administered with simvastatin compared with atorvastatin in adults with hypercholesterolemia. Am J Cardiol 2004;93(12):1487-94.
10. Barter PJ, O'Brien RC. Achievement of target plasma cholesterol levels in hypercholesterolaemic patients being treated in general practice. Atherosclerosis 2000;149(1):199-205.
11. Bays HE, Dujovne CA, McGovern ME, White TE, Kashyap ML, Hutcheson AG, et al. Comparison of once-daily, niacin extended-release/lovastatin with standard doses of atorvastatin and simvastatin (the ADvicor Versus Other Cholesterol-Modulating Agents Trial Evaluation [ADVOCATE]). Am J Cardiol 2003;91(6):667-72.
12. Betteridge DJ. International multicentre comparison of cerivastatin with placebo and simvastatin for the treatment of patients with primary hypercholesterolaemia. International Cerivastatin Study Group. Int J Clin Pract 1999;53(4):243-50.
13. Bilz S, Wagner S, Schmitz M, Bedynek A, Keller U, Demant T. Effects of atorvastatin versus fenofibrate on apoB-100 and apoA-I kinetics in mixed hyperlipidemia. J Lipid Res 2004;45(1):174-85.

14. Bleske BE, Willis RA, Anthony M, Casselberry N, Datwani M, Uhley VE, et al. The effect of pravastatin and atorvastatin on coenzyme Q10. Am Heart J 2001;142(2):E2.
15. Bo M, Nicolello MT, Fiandra U, Mercadante G, Piliego T, Fabris F. Treatment of heterozygous familial hypercholesterolemia: atorvastatin vs simvastatin. Nutr Metab Cardiovasc Dis 2001;11(1):17-24.
16. Borghi C, Dormi A, Veronesi M, Sangiorgi Z, Gaddi A. Association between different lipid-lowering treatment strategies and blood pressure control in the Brisighella Heart Study. Am Heart J 2004;148(2):285-92.
17. Branchi A, Fiorenza AM, Rovellini A, Torri A, Muzio F, Macor S, et al. Lowering effects of four different statins on serum triglyceride level. Eur J Clin Pharmacol 1999;55(7):499-502.
18. Branchi A, Fiorenza AM, Torri A, Muzio F, Berra C, Colombo E, et al. Effects of low doses of simvastatin and atorvastatin on high-density lipoprotein cholesterol levels in patients with hypercholesterolemia. Clin Ther 2001;23(6):851-7.
19. Brown WV, Bays HE, Hassman DR, McKenney J, Chitra R, Hutchinson H, et al. Efficacy and safety of rosuvastatin compared with pravastatin and simvastatin in patients with hypercholesterolemia: a randomized, double-blind, 52-week trial. Am Heart J 2002;144(6):1036-43.
20. Byington RP, Evans GW, Espeland MA, Applegate WB, Hunninghake DB, Probstfield J, et al. Effects of lovastatin and warfarin on early carotid atherosclerosis: sex-specific analyses. Asymptomatic Carotid Artery Progression Study (ACAPS) Research Group. Circulation 1999;100(3):e14-7.
21. Cannon CP, Braunwald E, McCabe CH, Rader DJ, Rouleau JL, Belder R, et al. Intensive versus moderate lipid lowering with statins after acute coronary syndromes. N Engl J Med 2004;350(15):1495-504.
22. Canudas J, Cenarro A, Civeira F, Garci-Otin AL, Aristegui R, Diaz C, et al. Chitotriosidase genotype and serum activity in subjects with combined hyperlipidemia: effect of the lipid-lowering agents, atorvastatin and bezafibrate. Metabolism 2001;50(4):447-50.
23. Capuzzi DM, Morgan JM, Carey CM, Intenzo C, Tulenko T, Kearney D, et al. Rosuvastatin alone or with extended-release niacin: a new therapeutic option for patients with combined hyperlipidemia. Prev Cardiol 2004;7(4):176-81.
24. Capuzzi DM, Morgan JM, Weiss RJ, Chitra RR, Hutchinson HG, Cressman MD. Beneficial effects of rosuvastatin alone and in combination with extended-release niacin in patients with a combined hyperlipidemia and low high-density lipoprotein cholesterol levels. Am J Cardiol 2003;91(11):1304-10.
25. Carlsson CM, Papcke-Benson K, Carnes M, McBride PE, Stein JH. Health-related quality of life and long-term therapy with pravastatin and tocopherol (vitamin E) in older adults. Drugs Aging 2002;19(10):793-805.
26. Carr-Lopez S, Exstrum T, Morse T, Shepherd M, Bush AC. Efficacy of three statins at lower maintenance doses. Clin Ther 1999;21(2):331-9.
27. Castano G, Mas R, Arruzazabala ML, Noa M, Illnait J, Fernandez JC, et al. Effects of policosanol and pravastatin on lipid profile, platelet aggregation and endothelemia in older hypercholesterolemic patients. Int J Clin Pharmacol Res 1999;19(4):105-16.

28. Castano G, Mas R, Fernandez L, Gamez R, Illnait J. Effects of policosanol and lovastatin in patients with intermittent claudication: a double-blind comparative pilot study. Angiology 2003;54(1):25-38.
29. Castano G, Mas R, Fernandez L, Illnait J, Mesa M, Alvarez E, et al. Comparison of the efficacy and tolerability of policosanol with atorvastatin in elderly patients with type II hypercholesterolaemia. Drugs Aging 2003;20(2):153-63.
30. Castano G, Menendez R, Mas R, Amor A, Fernandez JL, Gonzalez RL, et al. Effects of policosanol and lovastatin on lipid profile and lipid peroxidation in patients with dyslipidemia associated with type 2 diabetes mellitus. Int J Clin Pharmacol Res 2002;22(3-4):89-99.
31. Chan DC, Watts GF, Mori TA, Barrett PH, Beilin LJ, Redgrave TG. Factorial study of the effects of atorvastatin and fish oil on dyslipidaemia in visceral obesity. Eur J Clin Invest 2002;32(6):429-36.
32. Civeira F, Cenarro A, Ferrando J, Puzo J, Garcia-Otin AL, Mozas P, et al. Comparison of the hypolipidemic effect of gemfibrozil versus simvastatin in patients with type III hyperlipoproteinemia. Am Heart J 1999;138(1 Pt 1):156-62.
33. Cortellaro M, Cofrancesco E, Boschetti C, Cortellaro F, Mancini M, Mariani M, et al. Effects of fluvastatin and bezafibrate combination on plasma fibrinogen, t-plasminogen activator inhibitor and C reactive protein levels in coronary artery disease patients with mixed hyperlipidaemia (FACT study). Fluvastatin Alone and in Combination Treatment. Thromb Haemost 2000;83(4):549-53.
34. Crespo N, Illnait J, Mas R, Fernandez L, Fernandez J, Castano G. Comparative study of the efficacy and tolerability of policosanol and lovastatin in patients with hypercholesterolemia and noninsulin dependent diabetes mellitus. Int J Clin Pharmacol Res 1999;19(4):117-27.
35. Crouse JR, 3rd, Frohlich J, Ose L, Mercuri M, Tobert JA. Effects of high doses of simvastatin and atorvastatin on high-density lipoprotein cholesterol and apolipoprotein A-I. Am J Cardiol 1999;83(10):1476-7, A7.
36. Davidson M, Ma P, Stein EA, Gotto AM, Jr., Raza A, Chitra R, et al. Comparison of effects on low-density lipoprotein cholesterol and high-density lipoprotein cholesterol with rosuvastatin versus atorvastatin in patients with type IIa or IIb hypercholesterolemia. Am J Cardiol 2002;89(3):268-75.
37. Davidson MH, Palmisano J, Wilson H, Liss C, Dicklin MR. A multicenter, randomized, double-blind clinical trial comparing the low-density lipoprotein cholesterol-lowering ability of lovastatin 10, 20, and 40 mg/d with fluvastatin 20 and 40 mg/d. Clin Ther 2003;25(11):2738-53.
38. Davis SR, Goldstat R, Newman A, Berry K, Burger HG, Meredith I, et al. Differing effects of low-dose estrogen-progestin therapy and pravastatin in postmenopausal hypercholesterolemic women. Climacteric 2002;5(4):341-50.
39. de Lorgeril M, Salen P, Bontemps L, Belichard P, Geyssant A, Itti R. Effects of lipid-lowering drugs on left ventricular function and exercise tolerance in dyslipidemic coronary patients. J Cardiovasc Pharmacol 1999;33(3):473-8.
40. Deedwania PC, Hunninghake DB, Bays HE, Jones PH, Cain VA, Blasetto JW. Effects of rosuvastatin, atorvastatin, simvastatin, and pravastatin on atherogenic dyslipidemia in patients with characteristics of the metabolic syndrome. Am J Cardiol 2005;95(3):360-6.

41. Deighan CJ, Caslake MJ, McConnell M, Boulton-Jones JM, Packard CJ. Comparative effects of cerivastatin and fenofibrate on the atherogenic lipoprotein phenotype in proteinuric renal disease. J Am Soc Nephrol 2001;12(2):341-8.
42. Derosa G, Mugellini A, Ciccarelli L, Fogari R. Randomized, double-blind, placebo-controlled comparison of the action of orlistat, fluvastatin, or both an anthropometric measurements, blood pressure, and lipid profile in obese patients with hypercholesterolemia prescribed a standardized diet. Clin Ther 2003;25(4):1107-22.
43. Desideri G, Croce G, Tucci M, Passacquale G, Broccoletti S, Valeri L, et al. Effects of bezafibrate and simvastatin on endothelial activation and lipid peroxidation in hypercholesterolemia: evidence of different vascular protection by different lipid-lowering treatments. J Clin Endocrinol Metab 2003;88(11):5341-7.
44. Despres JP, Lemieux I, Salomon H, Delaval D. Effects of micronized fenofibrate versus atorvastatin in the treatment of dyslipidaemic patients with low plasma HDL-cholesterol levels: a 12-week randomized trial. J Intern Med 2002;251(6):490-9.
45. Ducobu J, VanHaelst L, Salomon H. Comparison of micronized fenofibrate and pravastatin in patients with primary hyperlipidemia. J Cardiovasc Pharmacol 2003;41(1):60-7.
46. Dujovne CA, Bays H, Davidson MH, Knopp R, Hunninghake DB, Stein EA, et al. Reduction of LDL cholesterol in patients with primary hypercholesterolemia by SCH 48461: results of a multicenter dose-ranging study. J Clin Pharmacol 2001;41(1):70-8.
47. Dujovne CA, Knopp R, Kwiterovich P, Hunninghake D, McBride TA, Poland M. Randomized comparison of the efficacy and safety of cerivastatin and pravastatin in 1,030 hypercholesterolemic patients. The Cerivastatin Study Group. Mayo Clin Proc 2000;75(11):1124-32.
48. Durrington PN, Tuomilehto J, Hamann A, Kallend D, Smith K. Rosuvastatin and fenofibrate alone and in combination in type 2 diabetes patients with combined hyperlipidaemia. Diabetes Res Clin Pract 2004;64(2):137-51.
49. Ehrenberg BL, Lamon-Fava S, Corbett KE, McNamara JR, Dallal GE, Schaefer EJ. Comparison of the effects of pravastatin and lovastatin on sleep disturbance in hypercholesterolemic subjects. Sleep 1999;22(1):117-21.
50. Faludi AA, Aldrighi JM, Bertolami MC, Saleh MH, Silva RA, Nakamura Y, et al. Progesterone abolishes estrogen and/or atorvastatin endothelium dependent vasodilatory effects. Atherosclerosis 2004;177(1):89-96.
51. Farnier M, Portal JJ, Maigret P. Efficacy of atorvastatin compared with simvastatin in patients with hypercholesterolemia. J Cardiovasc Pharmacol Ther 2000;5(1):27-32.
52. Fehr T, Kahlert C, Fierz W, Joller-Jemelka HI, Riesen WF, Rickli H, et al. Statin-induced immunomodulatory effects on human T cells in vivo. Atherosclerosis 2004;175(1):83-90.
53. Frost RJ, Otto C, Geiss HC, Schwandt P, Parhofer KG. Effects of atorvastatin versus fenofibrate on lipoprotein profiles, low-density lipoprotein subfraction distribution, and hemorheologic parameters in type 2 diabetes mellitus with mixed hyperlipoproteinemia. Am J Cardiol 2001;87(1):44-8.
54. Garcia-Otin AL, Civeira F, Aristegui R, Diaz C, Recalde D, Sol JM, et al. Allelic polymorphism -491A/T in apo E gene modulates the lipid-lowering response in combined hyperlipidemia treatment. Eur J Clin Invest 2002;32(6):421-8.

55. Gentile S, Turco S, Guarino G, Sasso CF, Amodio M, Magliano P, et al. Comparative efficacy study of atorvastatin vs simvastatin, pravastatin, lovastatin and placebo in type 2 diabetic patients with hypercholesterolaemia. Diabetes Obes Metab 2000;2(6):355-62.
56. Gibellato MG, Moore JL, Selby K, Bower EA. Effects of lovastatin and pravastatin on cognitive function in military aircrew. Aviat Space Environ Med 2001;72(9):805-12.
57. Giral P, Bruckert E, Jacob N, Chapman MJ, Foglietti MJ, Turpin G. Homocysteine and lipid lowering agents. A comparison between atorvastatin and fenofibrate in patients with mixed hyperlipidemia. Atherosclerosis 2001;154(2):421-7.
58. Gomez-Gerique JA, Ros E, Olivan J, Mostaza JM, Vilardell M, Pinto X, et al. Effect of atorvastatin and bezafibrate on plasma levels of C-reactive protein in combined (mixed) hyperlipidemia. Atherosclerosis 2002;162(2):245-51.
59. Gonbert S, Malinsky S, Sposito AC, Laouenan H, Doucet C, Chapman MJ, et al. Atorvastatin lowers lipoprotein(a) but not apolipoprotein(a) fragment levels in hypercholesterolemic subjects at high cardiovascular risk. Atherosclerosis 2002;164(2):305-11.
60. Haenni A, Ohrvall M, Lithell H. Serum magnesium status during lipid-lowering drug treatment in non-insulin-dependent diabetic patients. Metabolism 2001;50(10):1147-51.
61. Herrington DM, Werbel BL, Riley WA, Pusser BE, Morgan TM. Individual and combined effects of estrogen/progestin therapy and lovastatin on lipids and flow-mediated vasodilation in postmenopausal women with coronary artery disease. J Am Coll Cardiol 1999;33(7):2030-7.
62. Hognestad A, Aukrust P, Wergeland R, Stokke O, Gullestad L, Semb AG, et al. Effects of conventional and aggressive statin treatment on markers of endothelial function and inflammation. Clin Cardiol 2004;27(4):199-203.
63. Hunninghake D, Insull W, Knopp R, Davidson M, Lohrbauer L, Jones P, et al. Comparison of the efficacy of atorvastatin versus cerivastatin in primary hypercholesterolemia. Am J Cardiol 2001;88(6):635-9.
64. Hunninghake D, Insull W, Jr., Toth P, Davidson D, Donovan JM, Burke SK. Coadministration of colesevelam hydrochloride with atorvastatin lowers LDL cholesterol additively. Atherosclerosis 2001;158(2):407-16.
65. Hunninghake DB, Ballantyne CM, Maccubbin DL, Shah AK, Gumbiner B, Mitchel YB. Comparative effects of simvastatin and atorvastatin in hypercholesterolemic patients with characteristics of metabolic syndrome. Clin Ther 2003;25(6):1670-86.
66. Ikeda T, Sakurai J, Nakayama D, Takahashi Y, Matsuo K, Shibuya Y, et al. Pravastatin has an additional depressor effect in patients undergoing long-term treatment with antihypertensive drugs. Am J Hypertens 2004;17(6):502-6.
67. Illingworth DR, Crouse JR, 3rd, Hunninghake DB, Davidson MH, Escobar ID, Stalenhoef AF, et al. A comparison of simvastatin and atorvastatin up to maximal recommended doses in a large multicenter randomized clinical trial. Curr Med Res Opin 2001;17(1):43-50.
68. Inami S, Okamatsu K, Takano M, Takagi G, Sakai S, Sano J, et al. Effects of statins on circulating oxidized low-density lipoprotein in patients with hypercholesterolemia. Jpn Heart J 2004;45(6):969-75.

69. Inoue T, Hayashi M, Takayanagi K, Morooka S. Lipid-lowering therapy with fluvastatin inhibits oxidative modification of low density lipoprotein and improves vascular endothelial function in hypercholesterolemic patients. Atherosclerosis 2002;160(2):369-76.
70. Insull W, Kafonek S, Goldner D, Zieve F. Comparison of efficacy and safety of atorvastatin (10mg) with simvastatin (10mg) at six weeks. ASSET Investigators. Am J Cardiol 2001;87(5):554-9.
71. Ito Y, Kawasaki M, Yokoyama H, Okubo M, Sano K, Arai M, et al. Different effects of pravastatin and cerivastatin on the media of the carotid arteries as assessed by integrated backscatter ultrasound. Circ J 2004;68(8):784-90.
72. Jayaram S, Jain MM, Naikawadi AA, Gawde A, Desai A. Comparative evaluation of the efficacy, safety, and tolerability of rosuvastatin 10 mg with atorvastatin 10 mg in adult patients with hypercholesterolaemia: the first Indian study. J Indian Med Assoc 2004;102(1):48-50, 52.
73. Jialal I, Stein D, Balis D, Grundy SM, Adams-Huet B, Devaraj S. Effect of hydroxymethyl glutaryl coenzyme a reductase inhibitor therapy on high sensitive C-reactive protein levels. Circulation 2001;103(15):1933-5.
74. Jilma B, Joukhadar C, Derhaschnig U, Rassoul F, Richter V, Wolzt M, et al. Levels of adhesion molecules do not decrease after 3 months of statin therapy in moderate hypercholesterolaemia. Clin Sci (Lond) 2003;104(2):189-93.
75. Jones PH, Davidson MH, Stein EA, Bays HE, McKenney JM, Miller E, et al. Comparison of the efficacy and safety of rosuvastatin versus atorvastatin, simvastatin, and pravastatin across doses (STELLAR* Trial). Am J Cardiol 2003;92(2):152-60.
76. Jones PH, Hunninghake DB, Ferdinand KC, Stein EA, Gold A, Caplan RJ, et al. Effects of rosuvastatin versus atorvastatin, simvastatin, and pravastatin on non-high-density lipoprotein cholesterol, apolipoproteins, and lipid ratios in patients with hypercholesterolemia: additional results from the STELLAR trial. Clin Ther 2004;26(9):1388-99.
77. Joukhadar C, Klein N, Prinz M, Schrolnberger C, Vukovich T, Wolzt M, et al. Similar effects of atorvastatin, simvastatin and pravastatin on thrombogenic and inflammatory parameters in patients with hypercholesterolemia. Thromb Haemost 2001;85(1):47-51.
78. Kadikoylu G, Yukselen V, Yavasoglu I, Bolaman Z. Hemostatic effects of atorvastatin versus simvastatin. Ann Pharmacother 2003;37(4):478-84.
79. Karalis DG, Ross AM, Vacari RM, Zarren H, Scott R. Comparison of efficacy and safety of atorvastatin and simvastatin in patients with dyslipidemia with and without coronary heart disease. Am J Cardiol 2002;89(6):667-71.
80. Kastelein JJ, Isaacsohn JL, Ose L, Hunninghake DB, Frohlich J, Davidson MH, et al. Comparison of effects of simvastatin versus atorvastatin on high-density lipoprotein cholesterol and apolipoprotein A-I levels. Am J Cardiol 2000;86(2):221-3.
81. Kazama H, Usui S, Okazaki M, Hosoi T, Ito H, Orimo H. Effects of bezafibrate and pravastatin on remnant-like lipoprotein particles and lipoprotein subclasses in type 2 diabetes. Diabetes Res Clin Pract 2003;59(3):181-9.
82. Kent SM, Flaherty PJ, Coyle LC, Markwood TT, Taylor AJ. Effect of atorvastatin and pravastatin on serum C-reactive protein. Am Heart J 2003;145(2):e8.

83. Kinlay S, Timms T, Clark M, Karam C, Bilodeau T, Ridker PM, et al. Comparison of effect of intensive lipid lowering with atorvastatin to less intensive lowering with lovastatin on C-reactive protein in patients with stable angina pectoris and inducible myocardial ischemia. Am J Cardiol 2002;89(10):1205-7.
84. Koh KK, Cardillo C, Bui MN, Hathaway L, Csako G, Waclawiw MA, et al. Vascular effects of estrogen and cholesterol-lowering therapies in hypercholesterolemic postmenopausal women. Circulation 1999;99(3):354-60.
85. Koh KK, Schenke WH, Waclawiw MA, Csako G, Cannon RO, 3rd. Statin attenuates increase in C-reactive protein during estrogen replacement therapy in postmenopausal women. Circulation 2002;105(13):1531-3.
86. Kowalski J, Pawlicki L, Grycewicz J, Blaszczyk J, Irzmanski R, Ceglinski T, et al. Plasma antioxidative activity during atorvastatin and fluvastatin therapy used in coronary heart disease primary prevention. Fundam Clin Pharmacol 2004;18(1):93-6.
87. Lamendola C, Abbasi F, Chu JW, Hutchinson H, Cain V, Leary E, et al. Comparative effects of rosuvastatin and gemfibrozil on glucose, insulin, and lipid metabolism in insulin-resistant, nondiabetic patients with combined dyslipidemia. Am J Cardiol 2005;95(2):189-93.
88. Lanzarotto F, Panarotto B, Sorbara R, Panteghini M, Pagani F, Sosta S, et al. Effect of long term simvastatin administration as an adjunct to ursodeoxycholic acid: evidence for a synergistic effect on biliary bile acid composition but not on serum lipids in humans. Gut 1999;44(4):552-6.
89. Lau WC, Waskell LA, Watkins PB, Neer CJ, Horowitz K, Hopp AS, et al. Atorvastatin reduces the ability of clopidogrel to inhibit platelet aggregation: a new drug-drug interaction. Circulation 2003;107(1):32-7.
90. Leiter LA, Hanna K. Efficacy and safety of cerivastatin in primary hypercholesterolemia: a long term comparative titration study with simvastatin. Can J Cardiol 1999;15(5):545-55.
91. Lemay A, Dodin S, Turcot L, Dechene F, Forest JC. Estrogen/progesterone replacement versus pravastatin and their sequential association in hypercholesterolemic postmenopausal women. Maturitas 2001;40(3):247-57.
92. Lemieux I, Laperriere L, Dzavik V, Tremblay G, Bourgeois J, Despres JP. A 16-week fenofibrate treatment increases LDL particle size in type IIA dyslipidemic patients. Atherosclerosis 2002;162(2):363-71.
93. Lemieux I, Salomon H, Despres JP. Contribution of apo CIII reduction to the greater effect of 12-week micronized fenofibrate than atorvastatin therapy on triglyceride levels and LDL size in dyslipidemic patients. Ann Med 2003;35(6):442-8.
94. MacMahon M, Kirkpatrick C, Cummings CE, Clayton A, Robinson PJ, Tomiak RH, et al. A pilot study with simvastatin and folic acid/vitamin B12 in preparation for the Study of the Effectiveness of Additional Reductions in Cholesterol and Homocysteine (SEARCH). Nutr Metab Cardiovasc Dis 2000;10(4):195-203.
95. Magen E, Viskoper R, Mishal J, Priluk R, Berezovsky A, Laszt A, et al. Resistant arterial hypertension and hyperlipidemia: atorvastatin, not vitamin C, for blood pressure control. Isr Med Assoc J 2004;6(12):742-6.
96. Magnani G, Carinci V, Magelli C, Potena L, Reggiani LB, Branzi A. Role of statins in the management of dyslipidemia after cardiac transplant: randomized controlled

trial comparing the efficacy and the safety of atorvastatin with pravastatin. J Heart Lung Transplant 2000;19(7):710-5.
97. Malik J, Melenovsky V, Wichterle D, Haas T, Simek J, Ceska R, et al. Both fenofibrate and atorvastatin improve vascular reactivity in combined hyperlipidaemia (fenofibrate versus atorvastatin trial--FAT). Cardiovasc Res 2001;52(2):290-8.
98. Marz W, Wollschlager H, Klein G, Neiss A, Wehling M. Safety of low-density lipoprotein cholestrol reduction with atorvastatin versus simvastatin in a coronary heart disease population (the TARGET TANGIBLE trial). Am J Cardiol 1999;84(1):7-13.
99. Mauger JF, Couture P, Paradis ME, Lamarche B. Comparison of the impact of atorvastatin and simvastatin on apoA-I kinetics in men. Atherosclerosis 2005;178(1):157-63.
100. Mayer O, Jr., Simon J, Holubec L, Pikner R, Subrt I. Fenofibrate-induced hyperhomocysteinemia may be prevented by folate co-administration. Eur J Clin Pharmacol 2003;59(5-6):367-71.
101. McKenney JM, Jones PH, Adamczyk MA, Cain VA, Bryzinski BS, Blasetto JW. Comparison of the efficacy of rosuvastatin versus atorvastatin, simvastatin, and pravastatin in achieving lipid goals: results from the STELLAR trial. Curr Med Res Opin 2003;19(8):689-98.
102. McKenney JM, McCormick LS, Schaefer EJ, Black DM, Watkins ML. Effect of niacin and atorvastatin on lipoprotein subclasses in patients with atherogenic dyslipidemia. Am J Cardiol 2001;88(3):270-4.
103. McPherson R. Comparative effects of simvastatin and cholestyramine on plasma lipoproteins and CETP in humans. Can J Clin Pharmacol 1999;6(2):85-90.
104. McPherson R, Hanna K, Agro A, Braeken A. Cerivastatin versus branded pravastatin in the treatment of primary hypercholesterolemia in primary care practice in Canada: a one-year, open-label, randomized, comparative study of efficacy, safety, and cost-effectiveness. Clin Ther 2001;23(9):1492-507.
105. Melenovsky V, Malik J, Wichterle D, Simek J, Pisarikova A, Skrha J, et al. Comparison of the effects of atorvastatin or fenofibrate on nonlipid biochemical risk factors and the LDL particle size in subjects with combined hyperlipidemia. Am Heart J 2002;144(4):E6.
106. Melenovsky V, Wichterle D, Simek J, Malik J, Haas T, Ceska R, et al. Effect of atorvastatin and fenofibrate on autonomic tone in subjects with combined hyperlipidemia. Am J Cardiol 2003;92(3):337-41.
107. Miettinen TA, Gylling H, Lindbohm N, Miettinen TE, Rajaratnam RA, Relas H. Serum noncholesterol sterols during inhibition of cholesterol synthesis by statins. J Lab Clin Med 2003;141(2):131-7.
108. Milionis HJ, Kakafika AI, Tsouli SG, Athyros VG, Bairaktari ET, Seferiadis KI, et al. Effects of statin treatment on uric acid homeostasis in patients with primary hyperlipidemia. Am Heart J 2004;148(4):635-40.
109. Mitsios JV, Papathanasiou AI, Rodis FI, Elisaf M, Goudevenos JA, Tselepis AD. Atorvastatin does not affect the antiplatelet potency of clopidogrel when it is administered concomitantly for 5 weeks in patients with acute coronary syndromes. Circulation 2004;109(11):1335-8.

110. Mullen MJ, Wright D, Donald AE, Thorne S, Thomson H, Deanfield JE. Atorvastatin but not L-arginine improves endothelial function in type I diabetes mellitus: a double-blind study. J Am Coll Cardiol 2000;36(2):410-6.
111. Nissen SE, Tuzcu EM, Schoenhagen P, Brown BG, Ganz P, Vogel RA, et al. Effect of intensive compared with moderate lipid-lowering therapy on progression of coronary atherosclerosis: a randomized controlled trial. Jama 2004;291(9):1071-80.
112. Nissen SE, Tuzcu EM, Schoenhagen P, Crowe T, Sasiela WJ, Tsai J, et al. Statin therapy, LDL cholesterol, C-reactive protein, and coronary artery disease. N Engl J Med 2005;352(1):29-38.
113. Ohmichi M, Ikegami H, Kurachi H, Node K, Morishige K, Nishio Y, et al. Effects of bezafibrate and simvastatin on plasma lipoproteins in hypercholesterolemia resistant to hormone replacement therapy. Maturitas 2001;38(3):279-86.
114. Olsson AG, Eriksson M, Johnson O, Kjellstrom T, Lanke J, Larsen ML, et al. A 52-week, multicenter, randomized, parallel-group, double-blind, double-dummy study to assess the efficacy of atorvastatin and simvastatin in reaching low-density lipoprotein cholesterol and triglyceride targets: the treat-to-target (3T) study. Clin Ther 2003;25(1):119-38.
115. Olsson AG, Istad H, Luurila O, Ose L, Stender S, Tuomilehto J, et al. Effects of rosuvastatin and atorvastatin compared over 52 weeks of treatment in patients with hypercholesterolemia. Am Heart J 2002;144(6):1044-51.
116. Ozsener S, Sendag F, Koc T, Terek MC, Oztekin K, Bilgin O. A comparison of continuous combined hormone replacement therapy, HMG-CoA reductase inhibitor and combined treatment for the management of hypercholesterolemia in postmenopausal women. J Obstet Gynaecol Res 2001;27(6):353-8.
117. Paoletti R, Fahmy M, Mahla G, Mizan J, Southworth H. Rosuvastatin demonstrates greater reduction of low-density lipoprotein cholesterol compared with pravastatin and simvastatin in hypercholesterolaemic patients: a randomized, double-blind study. J Cardiovasc Risk 2001;8(6):383-90.
118. Paolisso G, Barbagallo M, Petrella G, Ragno E, Barbieri M, Giordano M, et al. Effects of simvastatin and atorvastatin administration on insulin resistance and respiratory quotient in aged dyslipidemic non-insulin dependent diabetic patients. Atherosclerosis 2000;150(1):121-7.
119. Paragh G, Torocsik D, Seres I, Harangi M, Illyes L, Balogh Z, et al. Effect of short term treatment with simvastatin and atorvastatin on lipids and paraoxonase activity in patients with hyperlipoproteinaemia. Curr Med Res Opin 2004;20(8):1321-7.
120. Pauciullo P, Borgnino C, Paoletti R, Mariani M, Mancini M. Efficacy and safety of a combination of fluvastatin and bezafibrate in patients with mixed hyperlipidaemia (FACT study). Atherosclerosis 2000;150(2):429-36.
121. Piperi C, Kalofoutis C, Skenderi K, Economidou O, Kalofoutis A. Beneficial effects of raloxifene and atorvastatin on serum lipids and HDL phospholipids levels of postmenopausal women. J Obstet Gynaecol 2004;24(4):414-9.
122. Pokrovskaia EV, Gratsianskii NA, Averkov OV, Slavina NN, Vaulin NA, Deev AD. [Non ST elevation acute coronary syndrome: early use of pravastatin or atorvastatin is associated with divergent changes of parameters of hemostasis]. Kardiologiia 2003;43(6):4-13.

123. Pokrovskaia EV, Vaulin NA, Gratsianskii NA, Averkov OV, Deev AD. [Markers of inflammation and platelet aggregation in patients with non ST elevation acute coronary syndrome treated with atorvastatin or pravastatin]. Kardiologiia 2003;43(1):7-18.
124. Portal VL, Moriguchi EH, Vieira JL, Schio S, Mastalir ET, Buffe F, et al. Comparison of the effect of two HMG CoA reductase inhibitors on LDL susceptibility to oxidation. Arq Bras Cardiol 2003;80(2):156-61, 150-5.
125. Prat H, Roman O, Pino E. [Comparative effects of policosanol and two HMG-CoA reductase inhibitors on type II hypercholesterolemia]. Rev Med Chil 1999;127(3):286-94.
126. Raal FJ, Marais AD, Klepack E, Lovalvo J, McLain R, Heinonen T. Avasimibe, an ACAT inhibitor, enhances the lipid lowering effect of atorvastatin in subjects with homozygous familial hypercholesterolemia. Atherosclerosis 2003;171(2):273-9.
127. Recto CS, 2nd, Acosta S, Dobs A. Comparison of the efficacy and tolerability of simvastatin and atorvastatin in the treatment of hypercholesterolemia. Clin Cardiol 2000;23(9):682-8.
128. Reiter M, Wirth S, Pourazim A, Baghestanian M, Minar E, Bucek RA. Statin therapy has no significant effect on skin tissue cholesterol: results from a prospective randomized trial. Clin Chem 2005;51(1):252-4.
129. Renders L, Mayer-Kadner I, Koch C, Scharffe S, Burkhardt K, Veelken R, et al. Efficacy and drug interactions of the new HMG-CoA reductase inhibitors cerivastatin and atorvastatin in CsA-treated renal transplant recipients. Nephrol Dial Transplant 2001;16(1):141-6.
130. Rosenson RS, Tangney CC, Schaefer EJ. Comparative study of HMG-CoA reductase inhibitors on fibrinogen. Atherosclerosis 2001;155(2):463-6.
131. Rosenson RS, Wolff D, Tangney CC. Statins reduce oxidized low-density lipoprotein levels, but do not alter soluble intercellular cell-adhesion molecule-1 and vascular cell-adhesion molecule-1 levels in subjects with hypercholesterolaemia. Clin Sci (Lond) 2004;106(2):215-7.
132. Rustemeijer C, Schouten JA, Voerman HJ, Hensgens HE, Donker AJ, Heine RJ. Pravastatin compared to bezafibrate in the treatment of dyslipidemia in insulin-treated patients with type 2 diabetes mellitus. Diabetes Metab Res Rev 2000;16(2):82-7.
133. Saito Y, Yamada N, Teramoto T, Itakura H, Hata Y, Nakaya N, et al. A randomized, double-blind trial comparing the efficacy and safety of pitavastatin versus pravastatin in patients with primary hypercholesterolemia. Atherosclerosis 2002;162(2):373-9.
134. Sakabe K, Fukuda N, Wakayama K, Nada T, Shinohara H, Tamura Y. Time course differences for statin-induced pleiotropic effects in hypercholesterolemic patients. Int J Cardiol 2004;94(1):111-7.
135. Sasaki S, Nakagawa M, Nakata T, Azuma A, Sawada S, Takeda K, et al. Effects of pravastatin on exercise electrocardiography test performance and cardiovascular mortality and morbidity in patients with hypercholesterolemia: Lipid Intervention Study in Kyoto. Circ J 2002;66(1):47-52.
136. Saunders E, Ferdinand K, Yellen LG, Tonkon MJ, Krug-Gourley S, Poland M. Efficacy and safety of cerivastatin and pravastatin in the treatment of primary hypercholesterolemia. J Natl Med Assoc 2000;92(7):319-26.

137. Sawayama Y, Shimizu C, Maeda N, Tatsukawa M, Kinukawa N, Koyanagi S, et al. Effects of probucol and pravastatin on common carotid atherosclerosis in patients with asymptomatic hypercholesterolemia. Fukuoka Atherosclerosis Trial (FAST). J Am Coll Cardiol 2002;39(4):610-6.
138. Sawayama Y, Tatsukawa M, Okada K, Maeda N, Shimizu C, Kikuchi K, et al. Association of Chlamydia pneumoniae antibody with the cholesterol-lowering effect of statins. Atherosclerosis 2003;171(2):281-5.
139. Sbarouni E, Flevari P, Kroupis C, Kyriakides ZS, Koniavitou K, Kremastinos DT. The effects of raloxifene and simvastatin on plasma lipids and endothelium. Cardiovasc Drugs Ther 2003;17(4):319-23.
140. Sbarouni E, Kroupis C, Kyriakides ZS, Koniavitou K, Kremastinos DT. Cell adhesion molecules in relation to simvastatin and hormone replacement therapy in coronary artery disease. Eur Heart J 2000;21(12):975-80.
141. Sbarouni E, Kyriakides ZS, Kremastinos DT. Effect of simvastatin on serum C-reactive protein during hormone replacement therapy. Am J Cardiol 2004;93(2):217-8.
142. Schaefer EJ, McNamara JR, Tayler T, Daly JA, Gleason JL, Seman LJ, et al. Comparisons of effects of statins (atorvastatin, fluvastatin, lovastatin, pravastatin, and simvastatin) on fasting and postprandial lipoproteins in patients with coronary heart disease versus control subjects. Am J Cardiol 2004;93(1):31-9.
143. Schneck DW, Knopp RH, Ballantyne CM, McPherson R, Chitra RR, Simonson SG. Comparative effects of rosuvastatin and atorvastatin across their dose ranges in patients with hypercholesterolemia and without active arterial disease. Am J Cardiol 2003;91(1):33-41.
144. Schuster H, Barter PJ, Stender S, Cheung RC, Bonnet J, Morrell JM, et al. Effects of switching statins on achievement of lipid goals: Measuring Effective Reductions in Cholesterol Using Rosuvastatin Therapy (MERCURY I) study. Am Heart J 2004;147(4):705-13.
145. Schwartz GG, Bolognese MA, Tremblay BP, Caplan R, Hutchinson H, Raza A, et al. Efficacy and safety of rosuvastatin and atorvastatin in patients with hypercholesterolemia and a high risk of coronary heart disease: a randomized, controlled trial. Am Heart J 2004;148(1):e4.
146. Schweitzer M, Tessier D, Vlahos WD, Leiter L, Collet JP, McQueen MJ, et al. A comparison of pravastatin and gemfibrozil in the treatment of dyslipoproteinemia in patients with non-insulin-dependent diabetes mellitus. Atherosclerosis 2002;162(1):201-10.
147. Sebestjen M, Boh M, Keber I. Simvastatin and pravastatin equally improve flow-mediated dilation in males with hypercholesterolemia. Wien Klin Wochenschr 2002;114(23-24):999-1003.
148. Sebestjen M, Zegura B, Keber I. Both cerivastatin and fenofibrate improve arterial vasoreactivity in patients with combined hyperlipidaemia. J Intern Med 2002;251(1):77-85.
149. See VY, Jr., DeNofrio D, Goldberg L, Chang G, Sasseen B, Kolansky DM, et al. Effect of atorvastatin on postcardiac transplant increase in low-density lipoprotein cholesterol reduces development of intimal hyperplasia and progression of endothelial dysfunction. Am J Cardiol 2003;92(1):11-5.

150. Seed M, Weir MR. Double-masked comparison of the quality of life of hypercholesterolemic men treated with simvastatin or pravastatin. International Quality of Life Multicenter Group. Clin Ther 1999;21(10):1758-70.
151. Seljeflot I, Tonstad S, Hjermann I, Arnesen H. Improved fibrinolysis after 1-year treatment with HMG CoA reductase inhibitors in patients with coronary heart disease. Thromb Res 2002;105(4):285-90.
152. Seljeflot I, Tonstad S, Hjermann I, Arnesen H. Reduced expression of endothelial cell markers after 1 year treatment with simvastatin and atorvastatin in patients with coronary heart disease. Atherosclerosis 2002;162(1):179-85.
153. Semb AG, van Wissen S, Ueland T, Smilde T, Waehre T, Tripp MD, et al. Raised serum levels of soluble CD40 ligand in patients with familial hypercholesterolemia: downregulatory effect of statin therapy. J Am Coll Cardiol 2003;41(2):275-9.
154. Smilde TJ, van Wissen S, Wollersheim H, Trip MD, Kastelein JJ, Stalenhoef AF. Effect of aggressive versus conventional lipid lowering on atherosclerosis progression in familial hypercholesterolaemia (ASAP): a prospective, randomised, double-blind trial. Lancet 2001;357(9256):577-81.
155. Smith DG, McBurney CR. An economic analysis of the Atorvastatin Comparative Cholesterol Efficacy and Safety Study (ACCESS). Pharmacoeconomics 2003;21 Suppl 1:13-23.
156. Stein DT, Devaraj S, Balis D, Adams-Huet B, Jialal I. Effect of statin therapy on remnant lipoprotein cholesterol levels in patients with combined hyperlipidemia. Arterioscler Thromb Vasc Biol 2001;21(12):2026-31.
157. Stein EA, Farnier M, Waldstreicher J, Mercuri M. Effects of statins on biomarkers of bone metabolism: a randomised trial. Nutr Metab Cardiovasc Dis 2001;11(2):84-7.
158. Stein EA, Strutt K, Southworth H, Diggle PJ, Miller E. Comparison of rosuvastatin versus atorvastatin in patients with heterozygous familial hypercholesterolemia. Am J Cardiol 2003;92(11):1287-93.
159. Strandberg TE, Feely J, Sigurdsson EL. Twelve-week, multicenter, randomized, open-label comparison of the effects of rosuvastatin 10 mg/d and atorvastatin 10 mg/d in high-risk adults: a DISCOVERY study. Clin Ther 2004;26(11):1821-33.
160. Strauss WE, Lapsley D, Gaziano JM. Comparative efficacy and tolerability of low-dose pravastatin versus lovastatin in patients with hypercholesterolemia. Am Heart J 1999;137(3):458-62.
161. Tamura A, Watanabe T, Nasu M. Effects of atorvastatin and pravastatin on malondialdehyde-modified LDL in hypercholesterolemic patients. Circ J 2003;67(10):816-20.
162. Tarraga Lopez PJ, Celada Rodriguez A, Cerdan Oliver M, Solera Albero J, Ocana Lopez JM, J.de. Miguel Clave Jd. Analisis coste-efectividad de atorvastatina frente a simvastatina como tratamiento hipolipemiante en pacientes hipercolesterolemicos en atencion primaria. Atencion Primaria 2001;27(1):18-24.
163. Taylor AJ, Kent SM, Flaherty PJ, Coyle LC, Markwood TT, Vernalis MN. ARBITER: Arterial Biology for the Investigation of the Treatment Effects of Reducing Cholesterol: a randomized trial comparing the effects of atorvastatin and pravastatin on carotid intima medial thickness. Circulation 2002;106(16):2055-60.
164. Trip MD, van Wissen S, Smilde TJ, Hutten BA, Stalenhoef AF, Kastelein JJ. Effect of atorvastatin (80 mg) and simvastatin (40 mg) on plasma fibrinogen levels and

on carotid intima media thickness in patients with familial hypercholesterolemia. Am J Cardiol 2003;91(5):604-6.
165. Tuncer M, Suleymanlar G, Ersoy FF, Yakupoglu G. Comparison of the effects of simvastatin and pravastatin on acute rejection episodes in renal transplant patients. Transplant Proc 2000;32(3):622-5.
166. Valkonen VP, Laakso J, Paiva H, Lehtimaki T, Lakka TA, Isomustajarvi M, et al. Asymmetrical dimethylarginine (ADMA) and risk of acute coronary events. Does statin treatment influence plasma ADMA levels? Atheroscler Suppl 2003;4(4):19-22.
167. van Dam MJ, Penn HJ, den Hartog FR, Kragten HA, Trip MD, Buirma RJ, et al. A comparison of the efficacy and tolerability of titrate-to-goal regimens of simvastatin and fluvastatin: a randomized, double-blind study in adult patients at moderate to high risk for cardiovascular disease. Clin Ther 2001;23(3):467-78.
168. van den Akker JM, Bredie SJ, Diepenveen SH, van Tits LJ, Stalenhoef AF, van Leusen R. Atorvastatin and simvastatin in patients on hemodialysis: effects on lipoproteins, C-reactive protein and in vivo oxidized LDL. J Nephrol 2003;16(2):238-44.
169. van Tits LJ, Smilde TJ, van Wissen S, de Graaf J, Kastelein JJ, Stalenhoef AF. Effects of atorvastatin and simvastatin on low-density lipoprotein subfraction profile, low-density lipoprotein oxidizability, and antibodies to oxidized low-density lipoprotein in relation to carotid intima media thickness in familial hypercholesterolemia. J Investig Med 2004;52(3):177-84.
170. van Wissen S, Smilde TJ, de Groot E, Hutten BA, Kastelein JJ, Stalenhoef AF. The significance of femoral intima-media thickness and plaque scoring in the Atorvastatin versus Simvastatin on Atherosclerosis Progression (ASAP) study. Eur J Cardiovasc Prev Rehabil 2003;10(6):451-5.
171. van Wissen S, Smilde TJ, Trip MD, de Boo T, Kastelein JJ, Stalenhoef AF. Long term statin treatment reduces lipoprotein(a) concentrations in heterozygous familial hypercholesterolaemia. Heart 2003;89(8):893-6.
172. van Wissen S, Trip MD, Smilde TJ, de Graaf J, Stalenhoef AF, Kastelein JJ. Differential hs-CRP reduction in patients with familial hypercholesterolemia treated with aggressive or conventional statin therapy. Atherosclerosis 2002;165(2):361-6.
173. Vigna GB, Donega P, Passaro A, Zanca R, Cattin L, Fonda M, et al. Post-prandial effects of gemfibrozil vs simvastatin in hypercholesterolemic subjects with borderline hypertriglyceridemia. Nutr Metab Cardiovasc Dis 1999;9(5):234-43.
174. Vigna GB, Donega P, Zanca R, Barban A, Passaro A, Pansini F, et al. Simvastatin, transdermal patch, and oral estrogen-progestogen preparation in early-postmenopausal hypercholesterolemic women: a randomized, placebo-controlled clinical trial. Metabolism 2002;51(11):1463-70.
175. Vivekananthan K, Mehra MR, Uber PA, DeGruiter H, Lavie CJ, Milani RV. Comparison of the efficacy and safety of pravastatin and simvastatin in heart transplantation. J Assoc Physicians India 2002;50(5):682-4.
176. Waehre T, Damas JK, Gullestad L, Holm AM, Pedersen TR, Arnesen KE, et al. Hydroxymethylglutaryl coenzyme a reductase inhibitors down-regulate chemokines and chemokine receptors in patients with coronary artery disease. J Am Coll Cardiol 2003;41(9):1460-7.

177. Wagner AM, Jorba O, Bonet R, Ordonez-Llanos J, Perez A. Efficacy of atorvastatin and gemfibrozil, alone and in low dose combination, in the treatment of diabetic dyslipidemia. J Clin Endocrinol Metab 2003;88(7):3212-7.
178. Wakatsuki A, Okatani Y, Ikenoue N. Effects of combination therapy with estrogen plus simvastatin on lipoprotein metabolism in postmenopausal women with type IIa hypercholesterolemia. Atherosclerosis 2000;150(1):103-11.
179. Wang TD, Chen WJ, Lin JW, Cheng CC, Chen MF, Lee YT. Efficacy of fenofibrate and simvastatin on endothelial function and inflammatory markers in patients with combined hyperlipidemia: relations with baseline lipid profiles. Atherosclerosis 2003;170(2):315-23.
180. Watanabe S, Fukumoto S, Takeuchi Y, Fujita H, Nakano T, Fujita T. Effects of 1-year treatment with fluvastatin or pravastatin on bone. Am J Med 2001;110(7):584-7.
181. Watts GF, Barrett PH, Ji J, Serone AP, Chan DC, Croft KD, et al. Differential regulation of lipoprotein kinetics by atorvastatin and fenofibrate in subjects with the metabolic syndrome. Diabetes 2003;52(3):803-11.
182. Welzig CM, Shin DG, Park HJ, Kim YJ, Saul JP, Galper JB. Lipid lowering by pravastatin increases parasympathetic modulation of heart rate: Galpha(i2), a possible molecular marker for parasympathetic responsiveness. Circulation 2003;108(22):2743-6.
183. Wierzbicki AS, Lumb PJ, Chik G, Crook MA. Comparison of therapy with simvastatin 80 mg and atorvastatin 80 mg in patients with familial hypercholesterolaemia. Int J Clin Pract 1999;53(8):609-11.
184. Wierzbicki AS, Lumb PJ, Chik G, Crook MA. High-density lipoprotein cholesterol and triglyceride response with simvastatin versus atorvastatin in familial hypercholesterolemia. Am J Cardiol 2000;86(5):547-9, A9.
185. Wierzbicki AS, Lumb PJ, Chik G, Crook MA. Fibrinogen response with simvastatin versus atorvastatin in familial hypercholesterolemia. Am J Cardiol 2001;87(3):338-40, A9.
186. Wiklund O, Mattsson-Hulten L, Hurt-Camejo E, Oscarsson J. Effects of simvastatin and atorvastatin on inflammation markers in plasma. J Intern Med 2002;251(4):338-47.
187. Wilmink HW, Twickler MB, Banga JD, Dallinga-Thie GM, Eeltink H, Erkelens DW, et al. Effect of statin versus fibrate on postprandial endothelial dysfunction: role of remnant-like particles. Cardiovasc Res 2001;50(3):577-82.
188. Wu CC, Sy R, Tanphaichitr V, Hin AT, Suyono S, Lee YT. Comparing the efficacy and safety of atorvastatin and simvastatin in Asians with elevated low-density lipoprotein-cholesterol--a multinational, multicenter, double-blind study. J Formos Med Assoc 2002;101(7):478-87.
189. Yokoyama I, Inoue Y, Moritan T, Ohtomo K, Nagai R. Impaired myocardial vasodilatation during hyperaemic stress is improved by simvastatin but not by pravastatin in patients with hypercholesterolaemia. Eur Heart J 2004;25(8):671-9.
190. Yu WC, Chen CH, Tsao HM, Ding YA. A randomized, double-blind comparison of cerivastatin and lovastatin for treatment of primary hypercholesterolemia. Zhonghua Yi Xue Za Zhi (Taipei) 2002;65(6):260-7.
191. Zachoval R, Gerbes AL, Schwandt P, Parhofer KG. Short-term effects of statin therapy in patients with hyperlipoproteinemia after liver transplantation: results of a randomized cross-over trial. J Hepatol 2001;35(1):86-91.

192. Zambon D, Ros E, Rodriguez-Villar C, Laguna JC, Vazquez M, Sanllehy C, et al. Randomized crossover study of gemfibrozil versus lovastatin in familial combined hyperlipidemia: additive effects of combination treatment on lipid regulation. Metabolism 1999;48(1):47-54.
